# Electrical Conduction Characteristic of a 2D MXene Device with Cu/Cr_2_C/TiN Structure Based on Density Functional Theory

**DOI:** 10.3390/ma13173671

**Published:** 2020-08-20

**Authors:** Lei Wang, Jing Wen, Yuan Jiang, Qiaofeng Ou, Lei Yu, Bang-Shu Xiong, Bingxing Yang, Chao Zhang, Yi Tong

**Affiliations:** 1 School of Information Engineering, Nanchang Hangkong University, Nanchang 330000, China; wenj@nchu.edu.cn (J.W.); jiangyuan@nchu.edu.cn (Y.J.); Ou.Qiaofeng@nchu.edu.cn (Q.O.); 70399@nchu.edu.cn (L.Y.); 42021@nchu.edu.cn (B.-S.X.); 2 State of Key Laboratory of Polyolefins and Catalysis, Shanghai 200062, China; yangbingxing1987@163.com; 3 Shanghai Institute of Technology, Shanghai 201418, China; 4 Shanghai Research Institute of Chemical Industry Co., Ltd., Shanghai 200062, China; zcsrici@163.com; 5 College of Electronic and Optical Engineering & College of Microelectronics, Nanjing University of Posts and Telecommunications, Nanjing 210023, China

**Keywords:** Cr_2_C, first-principle study, MXene, memristor

## Abstract

The electronic structure and the corresponding electrical conductive behavior of the Cu/Cr_2_C/TiN stack were assessed according to a newly developed first-principle model based on density functional theory. Using an additional Cr_2_C layer provides the metal-like characteristic of the Cu/Cr_2_C/TiN stack with much larger electrical conduction coefficients (i.e., mobility, diffusivity, and electrical conductivity) than the conventional Ag/Ti_3_C_2_/Pt stack due to the lower activation energy. This device is therefore capable of offering faster switching speeds, lower programming voltage, and better stability and durability than the memristor device with conventional Ti_3_C_2_ MXene.

## 1. Introduction

The recent prosperity of two-dimensional (2D) materials has excited considerable interest in a rapid-growth family of carbides and nitrides of transition metals known as MXenes [1]. MXenes materials, formed by selectively etching layers of sp elements from their corresponding three-dimensional (3D) MAX phases, show a variety of compositions and structures leading to the formation of a large and rapidly expanding family of 2D materials [2,3]. The chemical formula of MXenes is generally defined as M_n+1_X_n_T_x,_, where M is an early transition metal (e.g., Ti, V, Cr, Mo, Mn, Sc, Zr, Hf), X is N and/or C, T is a surface termination unit, and n = 1, 2, or 3 [4]. In addition to the selective acid etching of their MAX/non-MAX parents, where A indicates A-group elements (normally group 13 and 14 elements on the periodic table) [5], other advanced techniques such as bottom-up construction and chemical transformation have also been adopted to produce MXenes [6,7]. Since the discovery of the first MXene (Ti_3_C_2_T_x_) in 2011 [8], a myriad of MXene compositions have been reported and subjected to intensive study either from experimental or theoretical perspectives [9,10,11]. These encouraging experiments clearly reveal numerous exotic properties of MXene such as high negative zea-potential, functionalized surfaces, mechanical properties of transition metal carbides/nitrides, and high electrical conductivity [12]. Because of these advantageous features, MXenes have most recently received widespread applications including in energy storage, biosensors, printable antennas, and topological insulators [13,14,15,16]. In spite of this fascinating progress, the electronic properties of MXenes and their applications still remain relatively mysterious.

Another emerging application of MXenes arises from its potential as a functional layer inside the conventional memristor device using binary transition metal oxides [17,18,19]. Memristor, originally conceptualized by Chua in 1971 [20] and physically realized in 2008 [21], is considered the 4th fundamental electrical component in addition to the resistor, capacitor, and inductor. The unique attribute of memristor arises from its pinched hysteresis loop that exhibits a relationship between the applied voltage and resulting current, indicating various resistance states with respect to external excitations [22,23]. Such resistance states can be naturally considered as the binary data, whereby a memristor device exhibits the capability of storing and processing data at the same place, and it has been extensively employed to imitate biological neurons and synapse systems [24,25,26]. Such an attractive trait relies on its unique, non-volatile resistive switching (RS) characteristic that can vary between high and low resistive states. However, resistance states of the memristor devices usually fluctuate during different write cycles, even with the same external stimulus [27]. One novel approach to address this issue is to introduce an additional MXene (i.e., Ti_3_C_2_) layer into the silicon dioxide (SiO_2_)-based memristor device to improve its repeatable RS characteristic [28]. Nevertheless, the physical mechanism regarding the role of MXene in determining the RS performance of the memristor still remains unclear. Furthermore, current research is mainly focused on the conventional Ti_3_C_2_ MXene, while overlooking the practicality of other MXene materials (e.g., Cr_2_C) for memristor applications. To comprehend the influence of MXene on the RS mechanism of the memristor, and thus seek for more promising MXene contenders for better memristive performances, the electronic structure and conductive characteristics of the MXene Cr_2_C adopted for a memristor structure of copper (Cu)/MXene (Cr_2_C)/titanium nitride (TiN) is investigated here through a newly developed computational model based on density functional theory (DFT).

## 2. Methods

**Figure 1:**
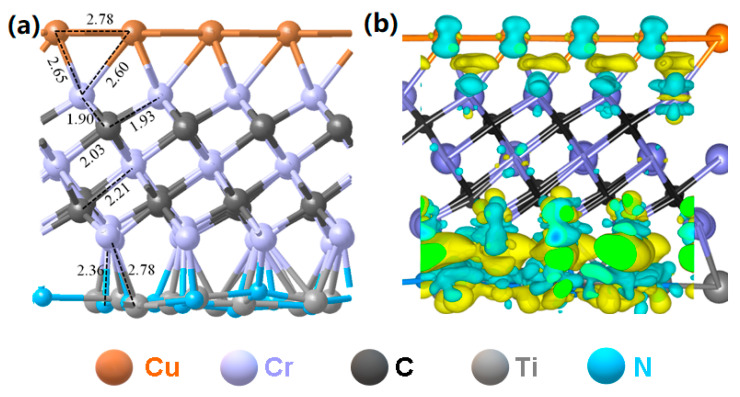
Calculated solid-state properties of Cu/Cr_2_C/TiN. (**a**) Structure and (**b**) charge redistribution (0.06 e/Å^3^). Color code: yellow and blue show partially negative charge and positive charge, respectively. The unit of bond length is Å.

All calculations were carried out using the Vienna ab initio simulation package (VASP) [29], and results were obtained using the MedeA^®^ software environment (Materials Design Inc; Santa Fe, NM, USA) [30]. The core–valence electron interaction was investigated by using the project-augmented wave (PAW) method [31,32], which is an extension of the augmented wave methods and the pseudo-potential approach. The Perdew, Burke, and Ernzerhof (PBE) functional [33] within the generalized gradient approximation (GGA) was employed to evaluate the electronic exchange and correlation energy. It should be pointed out that, compared with other functionals (such as Perdew-Wang 91, also known as PW91), PBE characterizes the exchange-correlation energy (E_xc_) with more smooth spatial variation and enhances the computational speed without sacrificing accuracy [34]. As a result, it has been commonly adopted for calculating the electronic structures of the MXene materials during the last decade [35,36]. However, it is speculated that using different functionals has a slight impact on the results. Cu(*3d*, *4s*), Cr(*3d*, *4s*), Ti(*3d*, *4s*), N(*2s*, *2p*), and C(*2s*, *2p*) were treated as valence electrons, corresponding to 11, 6, 4, 5, and 4 electrons in our calculations, respectively. The nudged elastic band (NEB) method was used to determine the transition states along the reaction pathways [37], and results were obtained using the MedeA^®^-Transition State Search module. For the Cr_2_C(001) surface slab, we used a five-layer thick model slab. To avoid lateral interactions, the calculations were carried out using a relatively large (4×4) supercell with a 15 Å vacuum gap. The Cu(111) and TiN(100) supported on Cr_2_C(001) is shown in Figure 1, and Cu(111) is on the top side of the Cr_2_C(001) surface while TiN(100) is located below the bottom side of Cr_2_C material. For all structural optimizations, all atoms were allowed to relax until the atomic forces reached below 0.05 eV/Å. The cutoff energy of the plane wave expansion was set to 400 eV, and a Monkhorst–Pack grid of 1 × 1 × 1 k-points was used because of the large size of the slab (a = b = 11.329 Å) [38].

## 3. Results and Discussion

A representative structure of the Cu/Cr_2_C/TiN surface is illustrated in Figure 1a. The Cu(111) and TiN(100) atomic layers can be stably combined with the Cr_2_C atomic layer, and both Cu(001) and TiN(100) surfaces bind with Cr atoms of Cr_2_C material. For TiN(100), both Ti and N ions bound to Cr, which caused structural change at the surface. Since Ti and N have different charges, N would be closer with Cr than Ti because of the electrostatic attraction. Such an effect resulted in a rippled interface between TiN(100) and Cr_2_C(001). We then calculated the charge density difference (Figure 1b), and most of the charge difference was at the two interfaces, i.e., Cu/Cr_2_C and Cr_2_C/TiN. For the Cu/Cr_2_C interface, most Cu and Cr atoms are positively charged, while the interstitial regions between the two layers are negatively charged. For the Cr_2_C/TiN interface, there was strong charge transfer between the two layers, and strong ionic Ti-N and Cr-N bonds are identified from Figure 1b.

**Figure 2:**
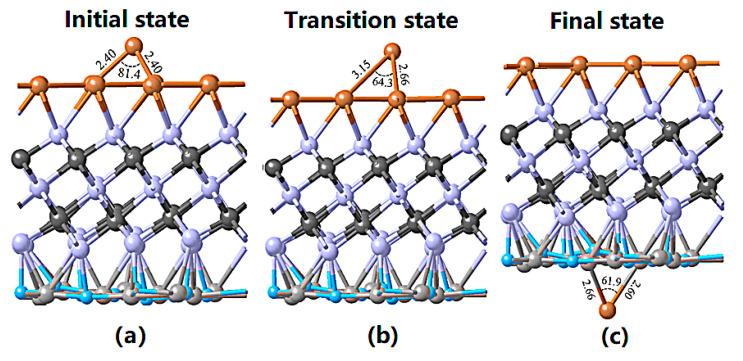
Cu diffusion through the Cu/Cr_2_C/TiN stack in its (**a**) initial state, (**b**) transition state, and (**c**) final state. The units of bond length and bond angle are angstrom (Å) and degree, respectively.

The resulting stronger charge distribution at the Cr_2_C/TiN interface seems to suggest that the Cr_2_C binds with TiN more strongly compared with Cu. As mentioned above, such an effect is due to the electrostatic attraction between the Cr and N atoms. The initial state, transition state, and final state structures of the Cu diffusion across the Cu/Cr_2_C/TiN structure are shown in Figure 2, respectively. A Cu atom was initially deposited at the vacuum side of the Cu(111) surface, and the Cu atom is expected to pass through the Cu(111) and Cr_2_C(100) surfaces. At the final state, Cu was bound at the vacuum side of the TiN surface. It is clear from Figure 2 that the transition state was similar to the initial state, with a calculated diffusion barrier of 0.67 eV. It can be inferred that the Cu atom can migrate inside the Cu/Cr_2_C/TiN structure more easily than diffusing from the vacuum to the Cu(111) surface. The difficulty of such diffusion at the Cu(111) surface may be attributed to the closely packed surface structure of Cu(111) where only tetrahedral holes can provide the ion-diffusion route.

**Figure 3:**
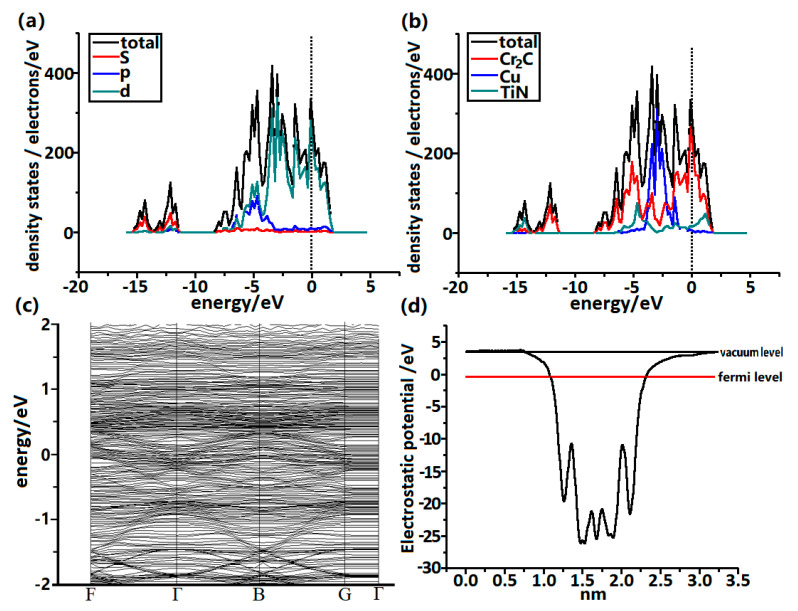
(**a**) Total density of states (TDOS) of Cu/Cr_2_C/TiN and partial density of states (PDOS) for *s*, *p*, and *d* orbitals. (**b**) TDOS of Cu/Cr_2_C/TiN and PDOS for Cr_2_C, Cu, and TiN. (**c**) Band structure of Cu/Cr_2_C/TiN. (**d**) Average static potential along Z-axis.

Total density of states (TDOS) and partial density of states (PDOS) of the Cu/Cr_2_C/TiN model were also studied. Figure 3a presents the TDOS and PDOS of *s*, *p*, and *d* orbitals, from which the Fermi energy level is mainly occupied by the valence electron of *d* orbitals. This can be understood since Ti, Cr, and Cu atoms were the main components of the system, and these three elements are all transition metals which give strong *d* band characteristics. The metallicity of the Cu/Cr_2_C/TiN was therefore evident without noticeable bandgap, suggesting good electrical conductivity. Figure 3b shows the TDOS and PDOS of Cr_2_C, Cu, and TiN, respectively. The strong hybridization between different atomic orbitals exhibited the bonding states among Cr_2_C, Cu, and TiN atomic layers, and in this case the Fermi energy level was mainly contributed by *d* orbitals of Cr_2_C. The band structure of the Cu/Cr_2_C/TiN model is illustrated in Figure 3c. Similar to the result of the TDOS and PDOS, the band structures showed no bandgap near the Fermi level, again demonstrating its large electrical conductivity. The work function, as plotted in Figure 3d, reveals that Cu(111) had higher work function than TiN(100), meaning that the potential of the electron at Cu(111) was higher than the counterpart of TiN(100). It can be predicted from the result that electrons may be preferable to transport from the Cu(111) side to the TiN(100) side.

The electronic behavior of the Cu/Cr2C/TiN stack was investigated by calculating its electrical conduction coefficients (i.e., mobility, diffusivity, and electrical conductivity) according to [39,40,41]

(1) $$\mu =(\frac{\delta^{2}\times v_{02}\times q}{6\times k_{B}\times T})\times \exp(\frac{-E_{a}}{k_{B}\times T}),$$

(2) $$D=\frac{\mu }{q}\times k_{B}\times T,$$

(3) $$\sigma =A\times \exp(\frac{-E_{a}}{k_{B}\times T})$$

where *μ* is the Cu atom mobility; *v*_02_ is the vibration frequency of Cu during the transitional state; *q* is the electronic charge; *T* is the temperature; *k_B_* is Bolzmann’s constant; *E_a_* is the activation energy for overcoming the diffusion barrier; *δ* is the migration distance of Cu; *D* is the diffusivity of Cu; and *σ* is the resulting electrical conductivity during the Cu diffusion. *A* is the pre-factor, given by

(4) $$A=\frac{k_{B}\times T}{h}\times \frac{Q^{TS}}{Q^{IS}}$$

where *Q^TS^* and *Q^IS^* area the partition functions per unit volume for a transition state and an initial state, respectively. Both *Q^TS^* and *Q^IS^* can be possessed from the calculated vibration partition function:

(5) $$Q_{v_{i}b}=\prod_{i}\frac{1}{1-e^{-h_{c}v_{i}/k_{B}T}}$$

where *c* and *v_i_* indicate the velocity of light and the vibrational frequency, respectively.

**Figure 4:**
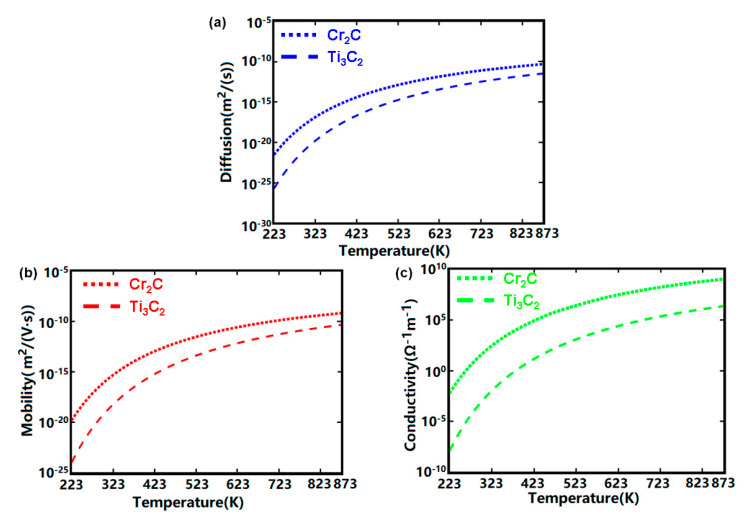
Electrical conduction coefficients of Cu atoms diffusing through Cu/Cr2C/TiN including (**a**) diffusivity, (**b**) mobility, and (**c**) electrical conductivity with respect to temperature.

**Figure 5:**
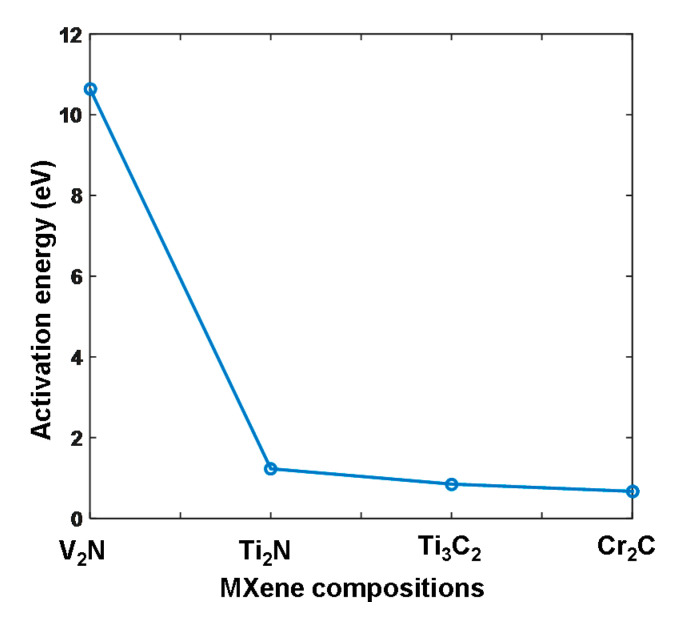
Calculated activation energies as a function of the designed stacks using different MXene compositions.

The Cu atom is assumed to migrate normally to the designed stack, and *δ* was therefore equal to the height of the stack in the z direction. The calculated temperature-dependent conduction coefficients of the Cu/Cr_2_C/TiN were subsequently compared with those of the Ag/Ti_3_C_2_/Pt stack (i.e., a typical memristive structure with MXene), as shown in Figure 4. According to Equations (1)–(3), the two key factors that affected the electronic performances of the Cu/Cr_2_C/TiN were *T* and *Ea*. It was found that increasing the temperature significantly increased the electrical conduction coefficients of the considered stack. Additionally, the calculated *Ea* of the Cu/Cr_2_C/TiN model was ~0.67 eV, lower than that of the Ti_3_C_2_ case (~0.85 eV). This provided the former stack with larger electrical conduction coefficients than the latter at the same temperature, which is also demonstrated in Figure 4. To further demonstrate its electrical conduction superiority, the aforementioned approaches were implemented to calculate *Ea* barriers of the designed stack (Cu/MXene/TiN) by using two other MXene media (i.e., V_2_N and Ti_2_N), and subsequently compared with the Ti_3_C_2_ and Cr_2_C cases, as illustrated in Figure 5. The calculated *Ea* barriers were found to be 10.64, 1.23, 0.85, and 0.67 eV for V_2_N, Ti_2_N, Ti_3_C_2_, and Cr_2_C, respectively. This implies that the designed stacks with Cr_2_C and V_2_N MXenes provided the strongest and weakest electrical conduction performances, respectively. It is therefore instructive to conceive that adding an extra Cr_2_C layer into the conventional memristor device, comprising one metal oxide layer sandwiched between Cu and TiN electrodes, can considerably accelerate the diffusion of the Cu atom inside the device and thus benefit the formation of a conductive filament (CF) along the locations of MXene nanostructures. This can remarkably improve the switching speed of the memristor and suppress the randomness of the CFs, thereby mitigating the stability and durability of the memristor device. Moreover, the resulting high electrical conductivity of the Cu/Cr_2_C/TiN stack is likely to lead to a lower programming voltage when compared to the Ti_3_C_2_ stack at the same temperature.

## 4. Conclusions

We performed first-principle calculations to predict the electronic structure and electrical conduction coefficients of the Cu/Cr_2_C/TiN stack based on density functional theory. Implementing the Cr_2_C layer endowed the designed stack with metal-like characteristics and much larger conduction coefficients (i.e., mobility, diffusivity, and electrical conductivity) than the conventional Ag/Ti_3_C_2_/Pt stack due to the lower activation energy. This device is therefore capable of offering faster switching speeds, lower programming voltage, and better stability and durability than the memristor device with conventional Ti_3_C_2_ MXene.
